# Congestion Based Mechanism for Route Discovery in a V2I-V2V System Applying Smart Devices and IoT

**DOI:** 10.3390/s150407768

**Published:** 2015-03-31

**Authors:** Natalia Parrado, Yezid Donoso

**Affiliations:** Grupo de Comunicaciones y Tecnologías de Información (COMIT), Departamento de Ingeniería de Sistemas y Computación, Universidad de Los Andes, Carrera 1Este 19ª-40, Bogotá 111711, Colombia; E-Mail: n.parrado31@uniandes.edu.co

**Keywords:** IoT, traffic congestion, vehicular routing, load balancing optimization, fairness, congestion, V2I, V2I, ITS

## Abstract

The Internet of Things is a new paradigm in which objects in a specific context can be integrated into traditional communication networks to actively participate in solving a determined problem. The Vehicle-to-Vehicle (V2V) and Vehicle-to-Infrastructure (V2I) technologies are specific cases of IoT and key enablers for Intelligent Transportation Systems (ITS). V2V and V2I have been widely used to solve different problems associated with transportation in cities, in which the most important is traffic congestion. A high percentage of congestion is usually presented by the inappropriate use of resources in vehicular infrastructure. In addition, the integration of traffic congestion in decision making for vehicular traffic is a challenge due to its high dynamic behavior. In this paper, an optimization model over the load balancing in the congestion percentage of the streets is formulated. Later, we explore a fully congestion-oriented route discovery mechanism and we make a proposal on the communication infrastructure that should support it based on V2I and V2V communication. The mechanism is also compared with a modified Dijkstra’s approach that reacts at congestion states. Finally, we compare the results of the efficiency of the vehicle’s trip with the efficiency in the use of the capacity of the vehicular network.

## 1. Introduction

With the constant expansion of cities due to the rapid economic and technological growth, there has been an increase in the population density and therefore, an increase in the density of motorized vehicles. The main effect of high vehicle densities in the cities is the phenomenon of congestion when a vehicle travelling to a destination shown unable to move because the number of moving vehicles exceeds the capacity of roads and blocks its route, so the vehicle has to wait until the vehicle ahead is able to move. The main effects of this waits are increments in travel times, fuel consumption, accident rates, and CO_2_ emissions (studies show that approximately 30% of carbon dioxide emissions generated by human activity is originated by vehicles [1]).

The main tactics to address the congestion problem in cities are: increase the number and size (capacity) of the existent roads, encourage people to use alternative to the automobile transport, selective and rationing use of roads through congestion charging systems [2] or using mechanisms such as the restriction of usage in specific time intervals [3], and use existing capacity of roads in a more efficiently way. From the above proposals, the first three focus on lighten the negative effects of congestion, but do not focus on the causes. The fourth proposal may have a greater impact because it focuses on how a vehicle is using the available streets. However, this is not a trivial approach since it is not easy for a driver to determine how his decisions are affecting the vehicular network state and therefore, his own trip. One of the main features of the congestion is that occurs in unpredictable ways, so there is no certainty on how many times the vehicle must wait and therefore, how much will be the total travel time based on these expectations.

The Department of Transportation of The United States created the Federal ITS Program, in which investment opportunities and initiatives are identified in any of these two aspects: transport infrastructure and vehicle applications; and classification of these projects has resulted in a taxonomy for existing ITS systems [4]. The Table 1 shows the current applications and if they focus on transport infrastructure or vehicular systems. 

**Table 1:** ITS taxonomy provided by [4].

	Infrastructure	Vehicles
Arterial Management	X	
Freeway Management	X	
Crash Prevention & Safety	X	
Road Weather Management.	X	
Roadway Operations & Maintenance	X	
Transit Management	X	
Traffic Incident Management	X	
Emergency Management	X	
Traveler Information	X	
Collision Avoidance		X
Driver Assistance		X
Collision Notification		X

Then, to achieve use the transport infrastructure efficiently, *Intelligent Transportation Systems* have been used in recent decades. The Intelligent Transportation Systems (ITS) are a set of new components and services that integrate information, infrastructure and communication technologies to allow the interaction of its elements and to provide specific functionality that improves the efficiency of urban transportation [5]. The field of ITS has expanded to support multiple operations, so there is now a large number of projects, proposals and applications. 

The most common applications for transport infrastructure are *Information Dissemination* applications, while in the set of applications for vehicular systems the most common are *Driver Assistance* applications. The former refers to systems that allow to collect and distribute information efficiently to vehicles and other elements that are part of the system, under specific traffic conditions. The second refers to applications that use information gathered from the context of each vehicle (such as speed or proximity to other vehicles), to allow a driver to make decisions in a timely manner to prevent accidents, improve the efficiency of its route, provide a safer journey, among others. 

**Figure 1:**
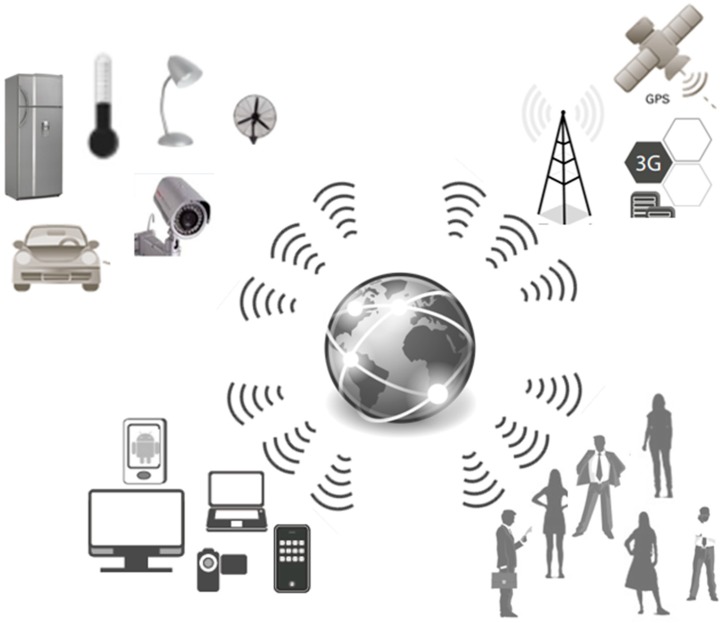
Internet of Things paradigm.

The solution methods rely on specific concepts such as the Internet of Things (IoT), whose paradigm is shown in Figure 1. IoT refers to any technology that allows the integration of different objects to the communications environment in such a way that the physic world could be accessed and managed through software. The main components involves traditional communication technologies (GSM, LTE, Wimax, *etc.*), and sensors and actuators in the target objects.

**Figure 2:**
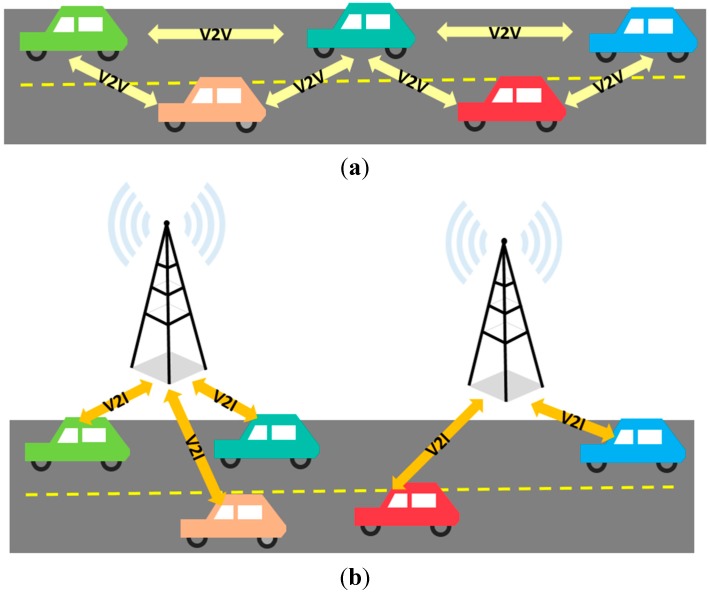
Representation of V2V and V2I technologies. (**a**) V2V communication; (**b**) V2I communication.

The concepts of vehicle-to-vehicle (V2V) communication and vehicle-to-infrastructure (V2I) communication have been introduced as subsets of IoT. V2V communication, shown in Figure 2a, allows two vehicles to interact directly without relying on a fixed communication infrastructure, while V2I communication, shown in Figure 2b, allows a vehicle to communicate with the elements of the road infrastructure such as traffic-light controllers, intelligent signalization, and others [6].

In the specific case of vehicle routing, traditional methods are based in static variables or the information derived from the road context is well known or calculated before the route is assigned to vehicles. This information usually does not change over time, for example, Dijsktra’s algorithm [7] based-approaches that focus in the minimal distance [8], or modified versions of Dijkstra that change the cost function to another parameters, *i.e.*, trip time, but assume they can be settled before the calculation of routes. In the case of congestion, this variable is not usually part of the optimization function in most of proposals, that means, developed methods are congestion aware but not congestion oriented. Moreover, when congestion must be taken into account, it is derived from other variables as expected time or velocity, but as mentioned before, those works can establish precalculated values to eliminate the dynamism in global congestion state ([9,10,11]). The reason of this is the high cost of maintaining and processing the changing congestion state in a way it is relevant for the decisions made. That’s why V2I and V2V systems are essential for addressing problems that involve high dynamic parameters. They allow the integration and collaboration of a high number of devices making the task of collecting and processing information of the context less complex.

The work in this paper was to develop a method in which condition to evaluate a route involves the definition of congestion in the path followed by the vehicle. Also, this method will be supported by a communication mechanism based on V2I or V2V communication allowing to address the problem of getting the most relevant global state of congestion in a totally dynamic environment. In addition, we evaluate the proposed method in terms of how efficient is the usage of available streets and propose an optimization model over the load balancing of the streets.

The rest of this paper is organized as follows: in Section 2 related work is discussed. In Section 3 the optimization model for load balancing of the use of capacity in each street is proposed. In Section 4 proposed mechanism of congestion-based is described as the supporting infrastructure. In Section 5 the simulation description, validation scenarios and results are presented. Finally in Section 6 we present the conclusions and future work.

## 2. Related Work

### 2.1. Technological Background for V2V and V2I Applications

Since the proposal of ITS in the decades of the 80’s and 90’s, these systems offer new ways to address the negative effects of the vehicular congestion problem in urban environments [12]. The work in ITS is based on the effective communication between different elements of the vehicular domain through new technologies.

**Figure 3:**
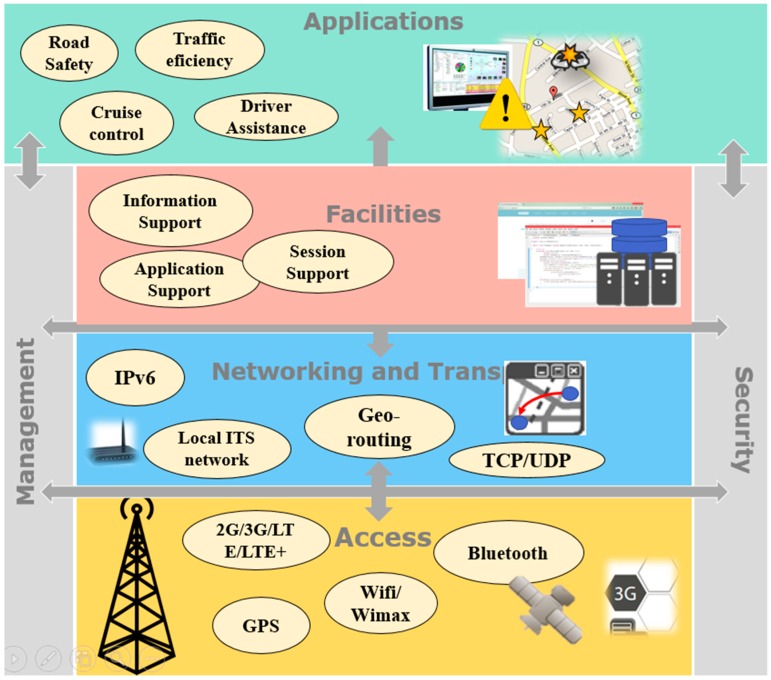
Main components in ITS systems architecture.

The European Telecommunication Standards Institute (ETSI) has defined an architecture standard for ITS communications (ITSC) [13]. The reference architecture follows the principle of the OSI model [14] defining an extension of the OSI layers to support ITS communication. As Figure 3 shows, three main layers are defined: *Access*, *Networking* and *Facilities*. In addition the *Applications*, *Management* and *Security* layers exist. The general architecture and example elements are shown below.

**Figure 4:**
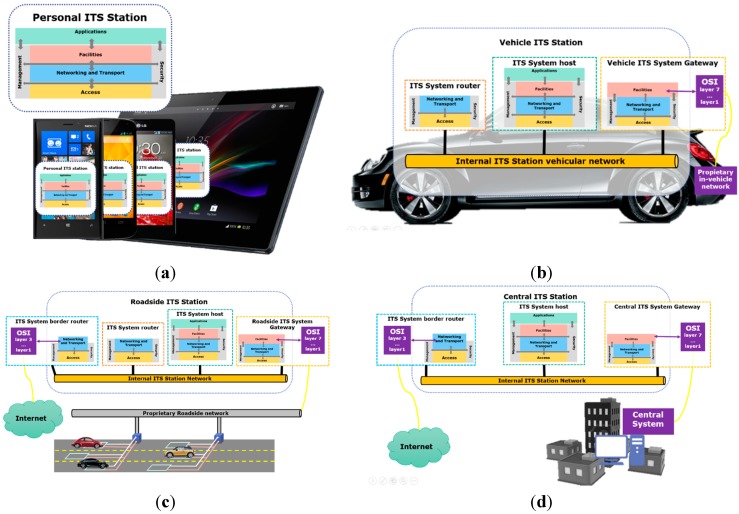
Existing ITS subsystems. (**a**) Personal ITS Subsystem; (**b**) Vehicle ITS subsystem; (**c**) Roadside ITS subsystem; (**d**) Central ITS Subsystem.

Three are the functional elements defined for ITSC: gateways, hosts and routers. This elements are present in several ITS ubsystems: personal ITS subsystem (Figure 4a), vehicle ITS subsystems (Figure 4b), roadside ITS subsystems (Figure 4c), and central ITS subsystems (Figure 4d). 

**Figure 5:**
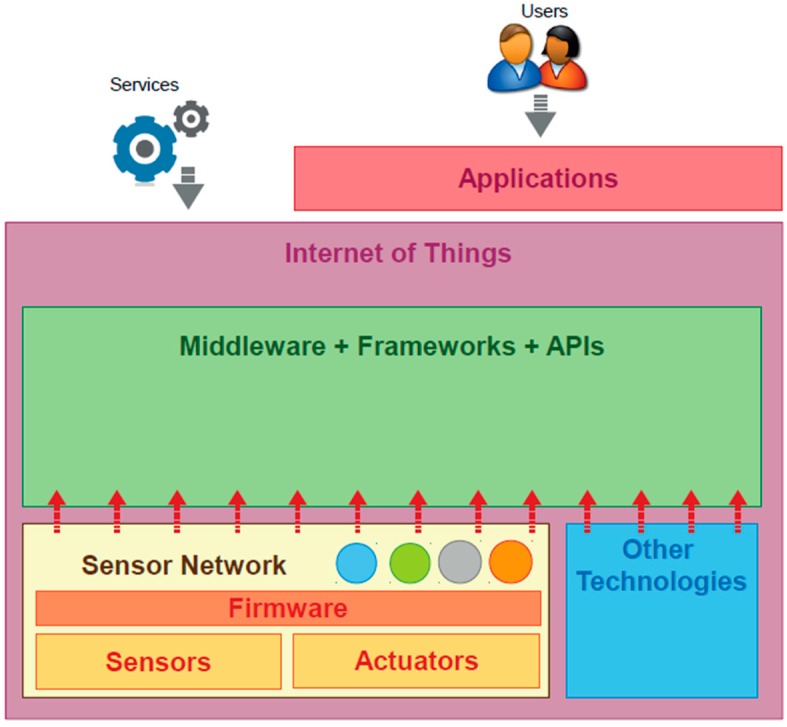
Relationship between sensor networks and IoT. Reproduced with permission from [15] Perera, C. *et al.*, IEEE Communications Surveys & Tutorials; published by IEEE, 2013.

The Internet of Things paradigm emerges as an enabler for ITS applications. IoT is totally based in the fact that objects in the real world can be integrated to traditional communications, serving as active components that are capable of modifying the context in which they locate, according to the needs of new specific services. The cornerstone in the IoT are the sensors and actuators available in the target objects which are incorporated to the communication architectures. Figure 5 shows the relationship between sensor networks and Internet of Things components [15].

Specific cases where sensor/actuators are used in combination with other network elements are Vehicle-to-Infrastructure and Vehicle-to-Vehicle communication. V2V and V2I are two subsets of Vehicular Ad-hoc Networks (VANETs), which are architectures for communication between two or more vehicles and between vehicles and road infrastructure, in which those who are equipped with processing capabilities and wireless communication capabilities can form networks as they move along the roads [16]. In the specification of the architecture for VANETs, some protocols are defined to achieving effective communication between elements in the network. 

In [16] is done a review of protocols in physical, MAC, network, transport and application. In the physical layer, Dedicated Short-Range Communication (DSRC) has been lately defined by different standardization entities to establish a short-range communication mechanism in moving vehicles (200 km/h and ranges from 300 to 1000 m). IEEE 802.11 technical committee have defined DSRC as 802.11p Wireless Access in Vehicular Environments (WAVE) [17]. Some researchers have focused in studying the performance of 802.11p in urban traffic environments [18,19,20]. On the other hand, 3GPP standards are used in other communication solutions proposed for V2V and V2I [21,22]. The comparison between these two technologies shows that the general performance of the communication between nodes where 802.11p is used is better than the performance obtained when the mechanism is based on 3GPP technologies (LTE for example) [23,24]. However the main advantage in the use of 3GPP technologies for vehicular communications relies in (1) the penetration rate in the market of devices that implement this technologies and (2) the existing deployed infrastructure. 

**Figure 6:**
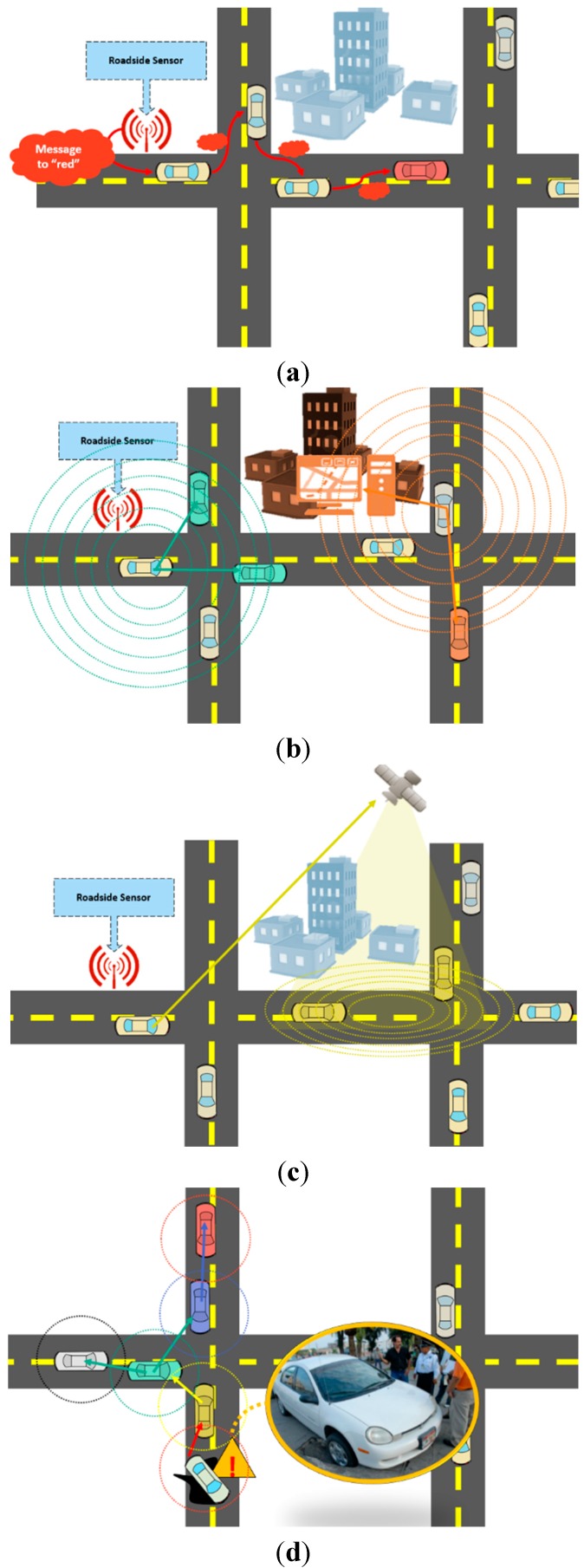
Supported mechanisms supported in vehicular networks. (**a**) Unicast communication; (**b**) Multicast communication; (**c**) Geocast communication; (**d**) Broadcast communication.

In addition, different communication mechanisms can be supported in vehicular networks [16]: Unicast Communication shown in Figure 6a, where data is sent from one source node to one target node via multi-hop wireless communication; Multicast/Geocast Communication shown in Figure 6b,c respectively, where in both, data is sent from one source node to multiple target nodes with the difference that in geocast communication the target nodes are grouped by geographical position; and Broadcast Communication shown in Figure 6d, where the source node sends data to all his neighbors.

### 2.2. Existing Dynamic Route Planning Algorithms

**Figure 7:**
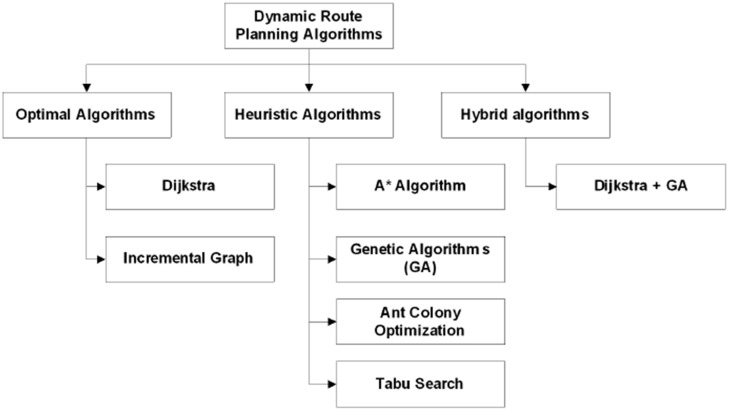
Classification of Dynamic Route Planning Algorithms. Reproduced with permission from [8] Djahel, S. *et al.*, First International Workshop on Vehicular Traffic Management for Smart Cities (VTM); published by IEEE, 2012.

In [8] a review of the most relevant algorithms to calculate routes in the vehicle routing stage of VPR problems is performed. These are classified into 3 groups as shown in Figure 7 [8].

Optimal Algorithms focus on finding the global optimal solution in the full range of existing solutions. In this category Dijkstra’s and incremental graph algorithms are found [25]. Heuristic Algorithms are algorithms that explore a subset of the total solution and allow to find a solution that is very close to the global optimum. As examples of heuristics algorithms we find A* Algorithm, Genetic algorithms [26], Ant Colony Optimization [27] and Tabu search [28]. Hybrid algorithms try to combine algorithms in each category to generate a powerful combined solution to compensate for any shortcomings or limitations on each individual strategy. As an example is the genetic algorithm for dynamic route planning using Dijkstra’s heuristic in multi-objective optimization, proposed by Kanoh *et al.* [29].

Based on the presented classification, some different contributions over the evaluation of the described algorithms are usually executed by authors in different scenarios. For example, in the work done by Wang *et al.* [30], four algorithms based on the shortest-path strategy are evaluated in a scenario with realistic data obtained from the TAPAS-Cologne project dataset, using SUMO simulator [31]. The algorithms evaluated were Dijkstra and A*, each one in a static and dynamic way. They select three areas (center, suburban and rural), and according to the evaluation performed, there is a suggestion for the most efficient routing algorithm in the different road scenarios.

## 3. Optimization Model for Load Balancing in Streets 

In this section we explore the proposal of a model of load balancing on congestion, so that resources (the streets with their respective capacities) are distributed among different users (vehicles) efficiently. In [32] a schema of multi-objective optimization following a MINMAX approach over the use of multicast routing links, *i.e.*, minimizing the maximum link utilization to get a better balance, is proposed. Additionally, in [33] Jain’s index [34] is used to describe the percentage of balance in the allocation of resources of heterogeneous wireless networks according to the network load. That is why we selected the equation proposed by Jain to describe the balance in the use of the streets of the vehicular network.

### 3.1. Vehicular Network Representation

**Figure 8:**
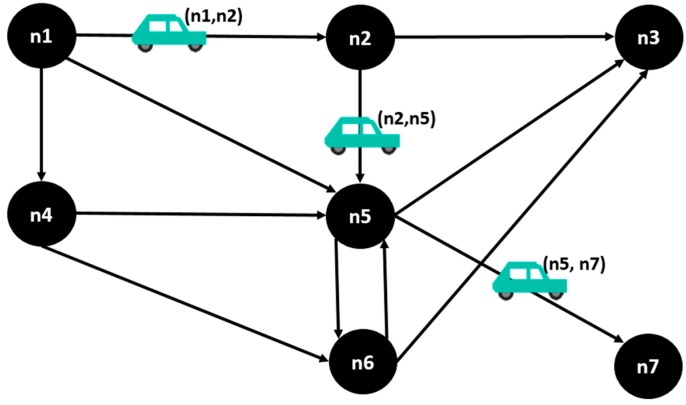
Illustration of a vehicle’s route in the vehicular network.

The vehicular network is modeled as a directed graph *G = (N*, *L)* where *N* is the set of nodes representing the intersections, and *L* is the set of links representing roads connecting the nodes; *i*, *j* *∈ N* are nodes and *(i*,*j)∈ L* is an ordered pair corresponding to a street *L*. For a vehicle v, nodes $e_{v},\;d_{v}\;\in N$ are defined, representing respectively, origin and destination node. A vehicle will move through the streets of the network until it reaches its destination, so its path corresponds to a set of streets $P_{v}=\{\left(e_{v},j_{1}\right),\ldots ,\;(i_{n},d_{v})\;\}$ as shown in the Figure 8.

**Figure 9:**
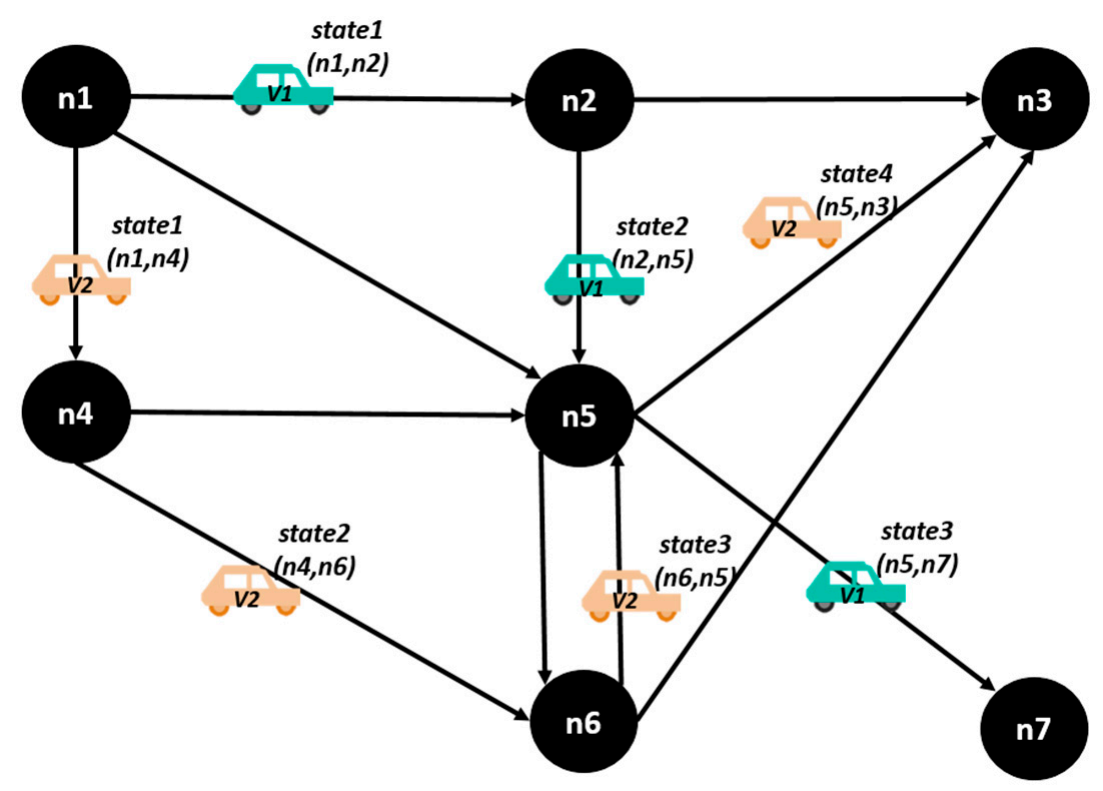
A vehicular network with four states.

The network has a set of states k corresponding to the way vehicles are located in the different network links, in certain moment of time. This states are illustrated in the Figure 9, where we have two vehicles moving through a vehicular network, and there are 4 total states: in the state 1, the **vehicle 1** is in the street (*n*1, *n*2), and the **vehicle 2** is in street (*n*1, *n*4); in the state 2, the **vehicle 1** is in (*n*2, *n*5) and the **vehicle 2** is in (*n*4, *n*6); in the state 3, **vehicle 1** is in (*n*5, *n*7) and the **vehicle 2** is in (*n*6, *n*5); in the state 4, **vehicle 1** is not in the network anymore, and **vehicle 2** is in (*n*5, *n*3).

A street (i,j) has an use $U_{\left(i,j\right)}(k)$ corresponding to the total number of vehicles on the street at a state k, and a maximum capacity$\;C_{\left(i,j\right)}$. A decision binary variable $X_{v\left(i,j\right)d_{v}}(k)$ is defined such that $X_{v\left(i,j\right)d_{v}}(k)$ = 1 if in a state k a vehicle v with destination $d_{v}$ takes the street (i,j), or 0 otherwise. Thus, the solution vector *X* is the set of assignments of each car to different streets of the network in each state.

### 3.2. Definition of Vehicular Congestion in the Optimization Model

The vehicular congestion in a street (i,j) is presented as the ratio between the use of the street at a state k, and the capacity of the street:

(1) $$\alpha_{\left(i,j\right)}\left(k\right)=\;\frac{U_{\left(i,j\right)}\left(k\right)}{C_{\left(i,j\right)}}=\;\frac{\sum_{v\in V}X_{v\left(i,j\right)d_{v}}\left(k\right)}{C_{\left(i,j\right)}}$$

### 3.3. Load Balancing Based on Jain’s Fairness Index

Jain’s fairness index equation is used to balance the congestion in each street of the vehicular network and it is applied to the formulated problem as follows:

(2) $$f_{\alpha }\left(k\right)=\frac{{\left[\sum_{\left(i,j\right)\in N}\alpha_{\left(i,j\right)}\left(k\right)\right]}^{2}}{\sum_{\left(i,j\right)\in N}Y_{\left(i,j\right)\;}*\sum_{\left(i,j\right)\in N}{\left(\alpha_{\left(i,j\right)}\left(k\right)\right)}^{2}},\;\alpha_{\left(i,j\right)}\;\geq 0\;\forall \left(i,\;j\right),\;\forall k$$

where $Y_{\left(i,j\right)}\;$is a binary variable = 1 if the nodes (i,  j) are connected, or 0 otherwise. 

The result for $f_{\alpha }(k)$ is in the range [0, 1], and a value closer to 1 represents a better assignation of the possible streets, so we can establish that the optimization model consists of maximizing the value for $f_{\alpha }\left(k\right)$.

### 3.4. Model Restrictions

The principal restrictions over the model presented above are:

#### 3.4.1. Capacity Restriction

The number of vehicles assigned to a street in a state *k* cannot exceed the capacity of the street:

(3) $$\underset{v\in V}{\sum }X_{v\left(i,j\right)d_{v}}\left(k\right)\;\leq C\left(i,\;j\right),\;\forall \left(i,j\right)\in N,\;\forall k\in K$$

#### 3.4.2. Connectivity Restriction

This constraint specifies that in the same state k a vehicle v cannot be assigned to two different streets:

(4) $$\underset{(i,j)\in N}{\sum }X_{v\left(i,j\right)d_{v}}\left(k\right)=max_{1\leq i,j\leq |N|}\left(X_{v\left(i,j\right)d_{v}}\left(k\right)\right)\;\forall v\in V,\;\forall k\in K$$

#### 3.4.3. Flow Restriction in Intermediate Nodes

In the intermediate nodes that are part of each vehicle’s route, the number of vehicles entering must equal the number of vehicles leaving, so that the sum of vehicle flows is equal to 0:

(5) $$\underset{k\;\in \;K}{\sum }\left(\underset{\left(i,j\right)\in N,\;}{\sum }X_{v\left(i,j\right)d_{v}}\left(k\right)-\underset{\left(i,j\right)\in N,\;v\;\in V}{\sum }X_{v\left(j,\;i\right)d_{v}}\left(k\right)\right)=0,\;i\neq e_{v},\;j\neq d_{v}\;\forall v\in V$$

#### 3.4.4. Destination Node

This constraint specifies that the path a vehicle takes should always end at its destination node:

(6) $$\underset{k\;\in \;K}{\sum }\underset{(i)\in N}{\sum }X_{v\left(i,j\right)d_{v}}\left(k\right)=1,\;j=d_{v},\forall v\in V$$

#### 3.4.5. Conclusions about the Used Model

**Figure 10:**
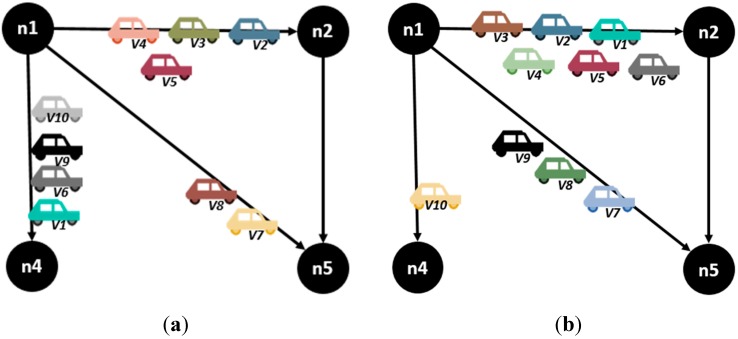
Obtained assignation using (**a**) the proposed model and (**b**) other optimization model based on route cost. (**a**) Obtained assignation with the proposed model (**b**) Obtained assignation with other model.

The model described was executed in the GAMS software, a general algebraic modeling tool used in linear, nonlinear and mixed integer optimization problems. Figure 10 shows the obtained results: in Figure 10a is the obtained assignation using the proposed model for a state *k* and in Figure 10b is the obtained assignation for a state *k* using an alternative model that optimizes the route cost. 

Table 2 shows calculated Fairness for each model.

**Table 2:** Results of assignation in Figure 10.

	C(*i, j*)	U(*i, j*) (a)	U(*i, j*) (b)	(a)	(b)
**n1.n2**	7	4	6	0.571429	0.857143
**n1.n4**	7	4	1	0.571429	0.142857
**n1.n5**	4	2	3	0.5	0.75
**FAIRNESS**				0.606403	0.442723

**Figure 11:**
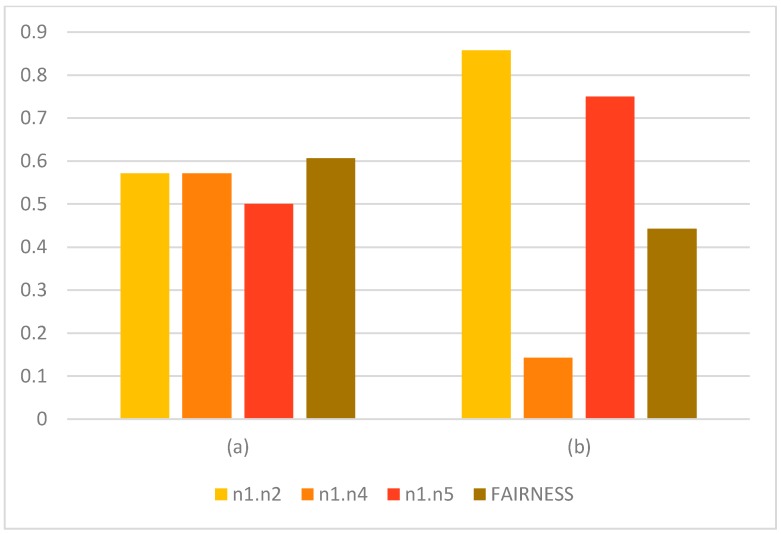
Graphical results in congestion level.

Figure 11 shows the usage of each link in each model and the obtained fairness.

**Figure 12:**
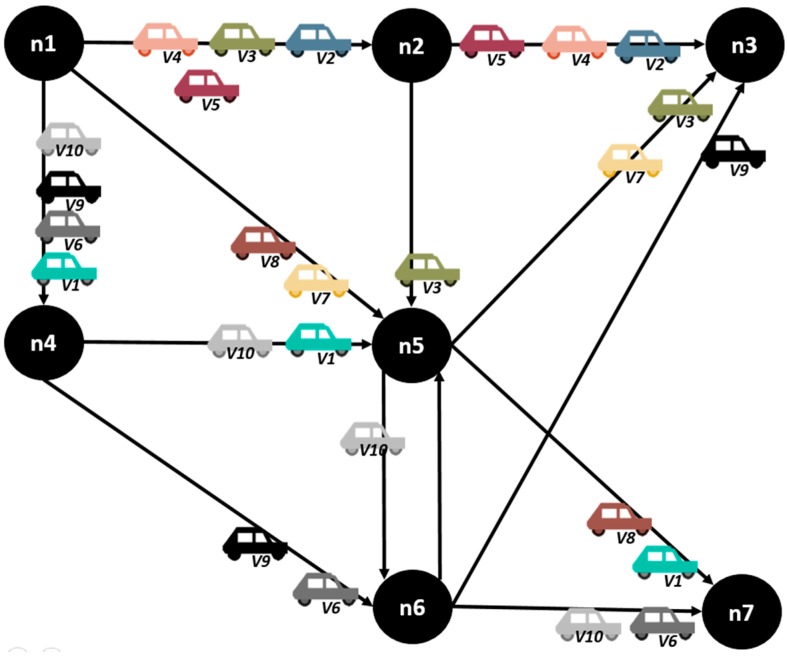
Overall assignation of the links.

As we see in the graphics above, the congestion levels are more balanced when the described model is used, which results in an increased value of fairness in the assignation of streets. Nevertheless, the assignation of streets showed for the entire route of vehicles in Figure 12 shows that the number of jumps is higher for some of the vehicles in order to achieve a better fairness value. In the next section, the proposal of a mechanism that optimizes the individual route of a vehicle is evaluated and compared with the fairness values obtained, so that the relevance of this metric in the vehicular network behavior is established.

## 4. Proposal

In several works we noticed that when considered, the congestion information of the vehicular networks is described locally and the global congestion state of the vehicular network cannot be established. This is a limitation on different methods to route vehicles and the reason is the extensive cost of determine and maintain the variable traffic information in an efficient way, mostly, if it is a central entity receiving the traffic information, and calculating and assigning routes to every different vehicle. That is the reason some authors have considered to integrate V2V and V2I communication methods, to reduce the amount of work needed to route a vehicle or react in congestion events. 

The work done in [35], is based in this idea. It establish an optimization model over average time in a vehicle’s route when the vehicle needs to be re-routed because of an unexpected congestion event, and uses a MAS architecture to implement the proposed solution and to assign the new route to a vehicle. In this case the optimization parameter referring to the traffic load is obtained locally. 

In this section, we explored a communication infrastructure that allows different elements in the vehicular network to interact to establish the information needed to obtain the best route based on global congestion. Additionally, we explain the executed algorithm based on the supporting infrastructure.

### 4.1. Communication Technologies and Elements in the Vehicular Network

The main elements communicating in the system are intersection nodes, vehicles and roadside sensors deployed over the road infrastructure. The vehicles are communication capable devices that also count with a GPS-enabled device in such a way the vehicle knows its actual position and the position of its destination node, and can communicate with other vehicles through the 802.11p technology. It also communicates with the intersection node for retrieving the information associated to its route and to report when the vehicle enters in a congestion state. The streets are seen as static elements with characteristics as distance and capacity, and the roadside sensors are capable of collecting data from the environment and transmit it to the intersection node (*i.e.*, vehicle counting and velocity measurements). 

**Figure 13:**
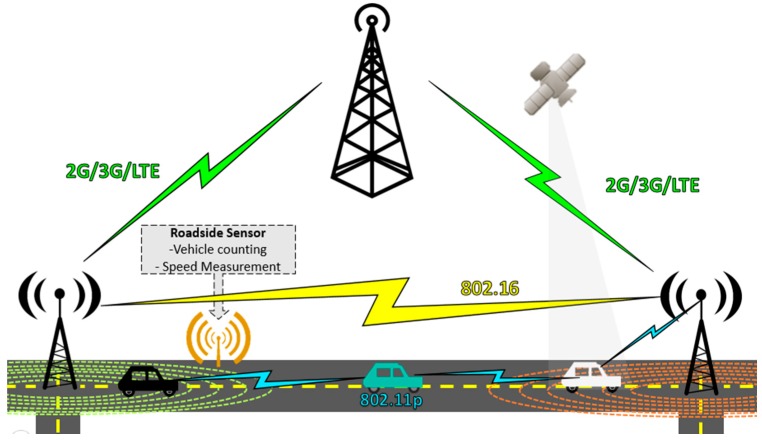
Supporting communication infrastructure.

**Figure 14:**
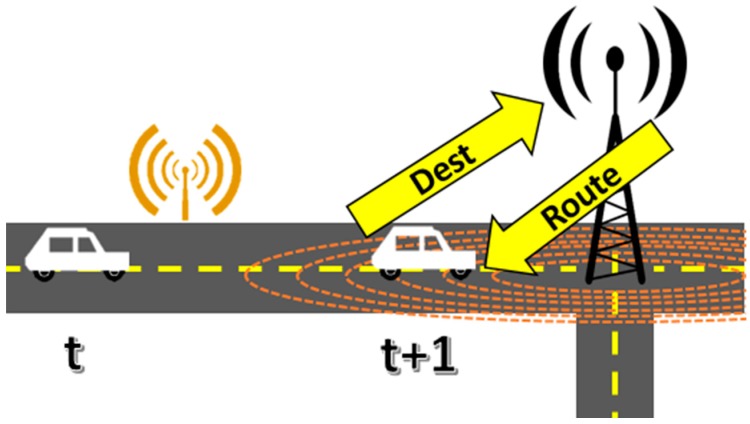
Communication between vehicle and intersection node.

**Figure 15:**
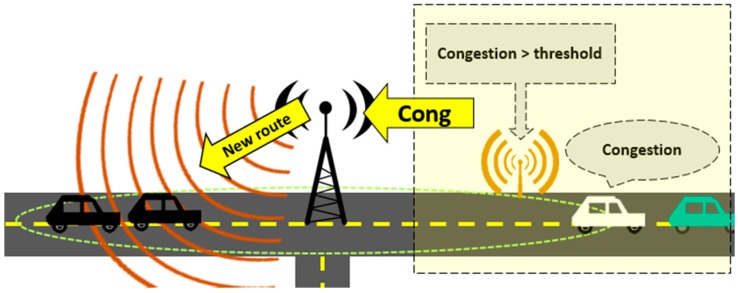
Route recalculation mechanism

Intersections nodes are seen as active elements and checkpoints, so are responsible for storing data collected from the roadside sensors to establish the congestion state, and can communicate with vehicles through 802.11p technology. Intersections also communicate with other intersections nodes forming *ad-hoc* networks through the 802.11p for short separation with the next node, or 802.16 for large separations, or through 3GPP communications as 2G, 3G and LTE. Figure 13 shows the supporting communication infrastructure:

The route assignment and recalculation service is going to be in each node, meaning that every intersection node is capable of assign the routes for vehicles in his neighborhood without relying in a central entity. The congestion data associated to the vehicular network is constructed through cooperation between nodes and roadside sensors, and it is shared among all nodes, so there is no need that a single entity recalculates the congestion state of the whole vehicular network every time a vehicle requests its route or affects the streets it uses. When a vehicle enters the range of the next intersection node, it transmits its route intentions and the intersection node assigns the set of streets the vehicle is going to follow according to the network congestion state, as shown in the Figure 14. Figure 15 shows the recalculation process: the recalculation service is triggered when the roadside sensors information reports the congestion exceeds a threshold, or when a vehicle reports it is unable to move. The recalculation message travels to the source intersection node in the street and that intersection node searches and assigns an alternative route to all vehicles in his range in the previous street. 

### 4.2. Proposed Algorithm

This section describes the main approach of the algorithm executed by the intersections to obtain the vehicle’s route or recalculate it if needed. Additionally, three more approximations are described in order to compare the proposed approach in terms of efficiency of the vehicle’s trip during the simulation executed in subsequent sections.

#### 4.2.1. Global Congestion Algorithm (GC)

The proposed solution aims to assign the route of a vehicle based on the congestion state reported in the different streets of the vehicular network, so when a vehicle decides to follow a route, it experiments the best global congestion state in subsequent streets. Therefore, the future approximated congestion for the vehicle is considered to get the best route the vehicle should follow. 

For this, a node has the information of potential routes and is able to get the congestion state for each one in such a way that the offered route is that with the less global congestion. That means that the vehicle is moving not just in the streets with less congestion, but in the path with less congestion, so the use of any street affects global congestion state of the path, even if is not the next connected street. In this way, we expected the algorithm would not just reacts to congestion (that means, only re-assigning routes when congestion is detected), but he could prevent congestion, reducing it from the first route assignation.

The potential routes are calculated before the execution of the algorithm, and the strategy is supported on the infrastructure that allows inter-communication node. This route calculation step generates an output that is a list of paths to any node in the topology. With that information, a node is capable to know all the existing paths to another node as long it exists. The route calculation method is not part of the scope of this paper. 

In addition, a node is capable of establish the global congestion state for each path exchanging messages with the nodes ahead, and it receives the recalculation messages triggered by the roadside sensors, or the vehicles ahead in its range. In both cases (a vehicle requests the route or a recalculation is needed), our route assign algorithm is executed by the intersection node to return the corresponding route for a vehicle.

The pseudo code for the Algorithm 1 is presented below.

**Algorithm 1.** Global congestion (GC)**Require:** Vehicle’s destination node d **Require:** All potential paths to d: $paths=\{p_{1},\;\ldots \;,p_{n}\}$ 1: Sort paths (A to Z) by $congestion_{p_{i}}$ 2: Path path=paths[0] 3: $path_{v}=path$ 4: **return**
$path_{v}$

The inputs to the algorithm are, the destination node to the vehicle and the path list of potential routes to the destination node. The idea is the algorithms only returns the path, and the assignation takes place for a requiring vehicle, or for a set of vehicles. In the case a vehicle requires the route, the vehicle informs the destination node to the intersection node and the algorithm is executed. In the case a recalculation is needed the intersection node requires for the vehicles in its range, their destination nodes. After, it generates the corresponding paths for each different destination node reported with the algorithm, and informs the vehicles the new path according with their route intention. This avoids the node has to repeat the algorithm for each vehicle when they have the same destination. 

#### 4.2.2. Modified Dijkstra Algorithm (MD)

This implementation was developed in order to compare what happens when a vehicle follows a route based on the distance, against the proposed congestion-based mechanism. In this case the initial route for any vehicle is calculated using Dijkstra heuristics to compute the shortest path for each vehicle. The vehicle will try to follow that path until it reaches the destination node. In the case the vehicle is incapable to advance to its next street, the intersection node recalculates the associated route using a Dijkstra’s strategy again, but only considering adjacent streets that are not congested (if any). 

This last change in finding alternative streets has the purpose of not replicate the congestion state for vehicles with the same origin/destination nodes. If a significant group have the same O/D characteristics and the route is not recalculated, the congestion could be always greater, making it very difficult to realize an objective comparison between the proposals.

In the absence of alternative roads, the vehicle has to wait in its current street. This last change in finding alternative streets has the purpose of not replicating the congestion state for vehicles with the same origin/destination nodes. Algorithm 2 shows the pseudo-code for the route recalculation process.

Like the original Dijkstra, the algorithm receives the destination node and have a distance matrix, but when it revises the next node with minimal distance (Algorithm 3), the algorithm verifies if the node is connected with the current node, and if so, if the street use is not equal to the capacity. That allows it to construct the shortest-route based on the next free streets, to get an alternative route so the car does not have to wait until its original route is free.

**Algorithm 2.** Modified Dijkstra Route Recalculation (MD)
**Require:** Requiring vehicle *v*
**Require:** Vehicle’s destination node *d*
**Require:** Actual node *actual*
**Require:** Adjacent nodes *Ad* = {*k*_1_*,…,k_n_*} not empty
**Require:** Adjacency distance matrix *d*[*n*] ▷*n = number of nodes*
**Require:** Un-visited nodes *unVisited* = { } empty
1: *d*[*actual*] = 0
2: add *actual* to *unVisited*
3: **while** Still are nodes in *unVisited* **do**
4: 	  *Min =* get minimal distance node (3)
5: 	  **if** *Min* does not exist **then**
6: 		**return** there is no available alternative route
7: 	  **else**
8: 		take *Min* from *unVisited*
9: 		**for all** *k* in adjacent nodes *Ad* = {*k*_1_*,…,k_n_*} **do**
10: 			**if** *d*[*k*] > *distance*_(*actual*,*k*)_ + *d*[*Min*] **then**
11: 				*d*[*k*] ← *distance*_(*actual*,*k*)_ + *d*[*Min*]
12: 			end if
13: 		end for
14: 	  **end if**
15: end while


**Algorithm 3.** Get minimal distance node
**Require:** Un-visited nodes *unVisited* = {*u*_1_,…,*u_n_*} not empty
**Require:** Actual node *actual*
**Require:** Adjacency distance matrix *d*[*n*]
1: *min* = *NotExists*
2: **for** *u* in *unVisited* **do**
3: 	  **if** *use*_(*actual*,*u*)_ ≥ *capacity*_(*actual*,*u*)_ *AND u* is not directly 
		  connected to *actual* **then**
4: 	   	take *u* from *unVisited*
5: 	  **else**
6: 	   	**if** *d*[*u*] < *d*[*min*] **then**
7: 			*min* ← *u*
8:	  	end if
9: 	  end if
10: end for
11: **return** *min*


#### 4.2.3. Local-Congestion Based Algorithm (LC)

In this approach a route was assigned to a vehicle based only in the local congestion information of the adjacent streets to the node. This was done in order to establish if the congestion information granted from the state of the neighborhood of a vehicle was enough to get good results, or if instead it is necessary to develop an exhaustive solution in the search space of the optimal. In this implementation, the intersection evaluates only its adjacent streets according to their congestion state, and reports to the vehicle the one with the less congestion between the available streets, that is, between those with sufficient space for the vehicle. The pseudo code is shown below (Algorithms 4 and 5).

**Algorithm 4.** Local Congestion (LC)
**Require:** Requiring vehicle *v*
**Require:** Actual node *actual*
1: Node *k* = Get next node in which congestion is minimal and has 
   space for the vehicle (6)
2: **if** *k* exists **then**
3: 	  **return** next street (*actual,k*)
4: **else** 
5:	  **return** there is no available route 
6: end if


**Algorithm 5.** Get next node
**Require:** Adjacent nodes *Ad* = {*k*_1_,…,*k_n_*} not empty
**Require:** Actual node *actual*
1: *min* = *NotExists*
2: **for** *k* in *Ad* **do**
3: 	  **if** *use*_(*actual*,*k*)_ < *capacity*_(*actual*,*k*)_ *AND*
                           *congestion*_(*actual*,*k*)_ < *congestion*_(*actual*,*min*)_
4: 	  	*min* ← *k*
5: 	  end if
6: end for
7: **return** *min*


#### 4.2.4. Natural Behavior Algorithm

In this implementation the objective was to model what would a vehicle do when moving around the city if the decision is not supported by any communication system. The vehicle is not communicating with the intersection nodes, so when it arrives to an intersection it just evaluate the streets adjacent to its current street and take the first whose capacity is less than its use. In the real live, this behavior is translated to drivers moving in the first in which they fit. The pseudo code is presented below (Algorithms 6 and 7), and is executed by the vehicle in the simulation.

**Algorithm 6.** Natural behavior
**Require:** Actual node *actual*
**Require:** Destination node *destination*
1: Node *k* = get next node that connects the first available street (7)
2: **if** *k* exists **then**
3: 	  **if** *k* = *destination* **then**
4: 	  	exit network 
5: 	  **else** 
6:		wait
7: 	  end if 
8: else
9: 	  wait
10: end if


**Algorithm 7.** Get first available node
**Require:** Actual node *actual*
**Require:** Adjacent nodes *Ad* = {*k*_1_,…,*k_n_*} not empty
1: **for** *k* in *Ad* **do**
2: 	  **if** *use*_(*actual*,*k*)_ < *capacity*_(*actual*,*k*)_ **then**
3: 	  	**return** *k*
4: 	  end if
5: end for
6: **return** *not Exists*


## 5. Validation Scenarios

This section describes how the validation process for the different approaches was. We defined a computational experiment based in two scenarios, a control scenario for small-scale examination of the results of each implementation, and a reference scenario that includes some features extracted from Google Maps. Additionally, the executed simulation is described and the evaluation metrics are specified for comparison between implementations.

### 5.1. Scenarios Definition

#### 5.1.1. Control Scenario

**Figure 16:**
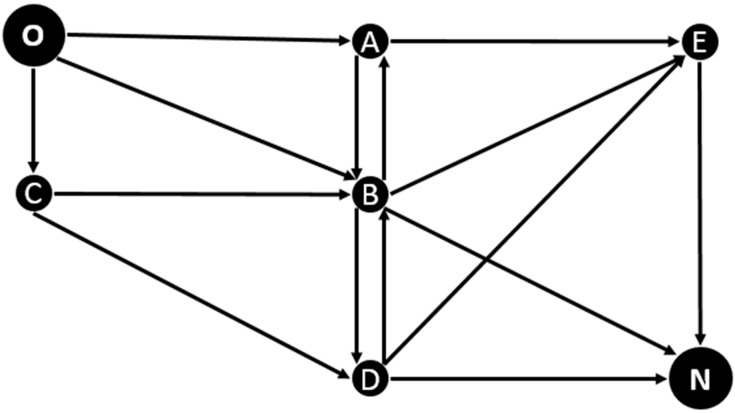
Proposed control scenario.

For the control scenario we had a small vehicular network composed by 15 streets with random capacities in the interval [7,20] and a distance equal to that capacity. Additionally, we defined specific origin and destination nodes O, N. The same O/D is selected to evaluate if when a group of vehicles with the same origin / destination characteristics gets a new route, and the new route for those vehicles is the same, congestion is moved to the new route instead of get better. Two different ways of simulation were developed here: in the first we established a maximum number N of vehicles moving through the network and entering in the same turn in order to establish how the vehicular network state evolves as the number of vehicles gets larger; in the second a number F of vehicles was incorporated after X steps in the simulation, to compare different flows of vehicles. The corresponding configuration is shown in Figure 16:

#### 5.1.2. Reference Scenario

**Figure 17:**
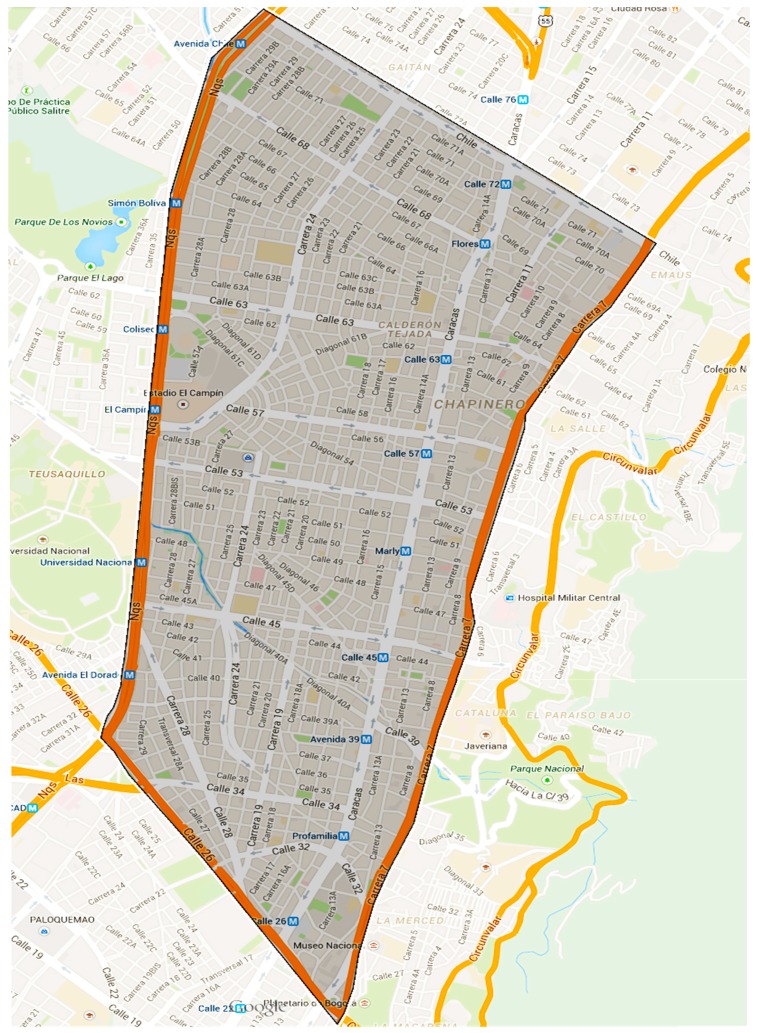
Desired congestion area, taken from Google Maps.

This scenario is based on the previous work realized in [2], which makes a proposal for congestion charging. This paper analyzed congestion in Colombian cities taking as a case study Bogota. As part of this work, areas of congestion were identified in the city of Bogotá which are located in the expanded center where the main economic activities of the city are concentrated. One of the identified areas was selected to create the reference scenario. Its main characteristics are shown in Figure 17.

**Figure 18:**
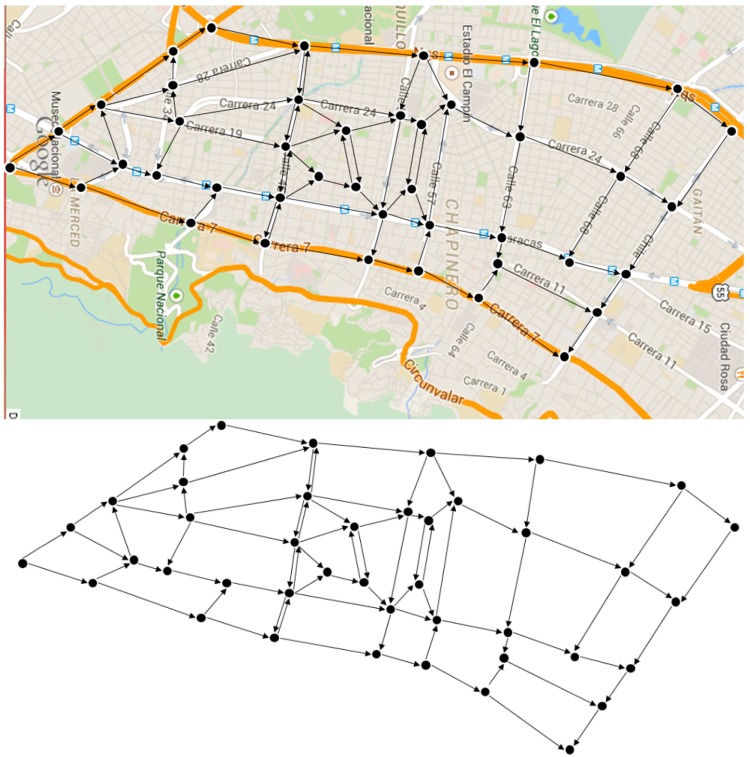
Proposed vehicular network.

Figure 18 shows the vehicular network created for this area. The network posed does not intend to be an accurate representation of this area, so it only aims to map the main corridors, with some adjustments to get more connected nodes, and the traffic flow direction was decided by the author. Based on the proposed vehicular network graph, it was necessary to establish the capacity of each street, the approximate number of vehicles to go through them and their respective origins and destinations.

##### (a) Street Capacity

To establish the capacity of each street, equation below was used:

(7) $$capacity_{i}=\;\frac{length\;street_{i}}{average\;length}$$

The average length is defined as the floor function of average length of a vehicle coupled with a minimum separation. This minimum corresponds to what is defined as spacing in the Colombia’s National Traffic Code [36], that is, the distance between two consecutive vehicles measured from the front end of a vehicle to the rear end of the other.

- For $length\;street_{i}$, approximate distances that provides Google Maps for each street network are taken.
- For average length=vehicle average length+min gap, is taken as an average length for all vehicles 3.8 m and 2.4 m of vehicle separation, so that the total is 6.2 m.

##### (b) Origin/Destination Pairs for Each Vehicle and Approximate Flow

In this case the vehicles depart each of a different origin and destination. Since we defined the flow of each street in the network, it was necessary to establish the subset of possible origins and destinations (O/D) so that any vehicle can reach its destination node. The O/D of a vehicle is assigned randomly of that set when the vehicle enters the network. Additionally, it was established a number *F* of vehicle incorporating in the network at each step of the simulation, thus, allowing a random number of vehicles joining each time. This is done in order to establish the robustness of the proposed solution because under different trip conditions, overall state of the network could change and it is useful to examine how the solution behaves under uncontrolled parameters.

### 5.2. Simulation Description

Since we didn’t used a specialized vehicular traffic simulator, it was necessary to model the behavior of a vehicle while it is moving in the vehicular network under certain assumptions. The vehicles are going to have three possibilities when following a route: move, wait and exit the network. When the vehicle starts its trip, it moves along the initial street in such a way that its final position never exceede the front vehicle position minus the minimal separation. If the separation is exceeded, the vehicle does not move and waits. The position changes of the vehicle are totally discrete. The flow diagram for the vehicle movement is shown in the figure below:

**Figure 19:**
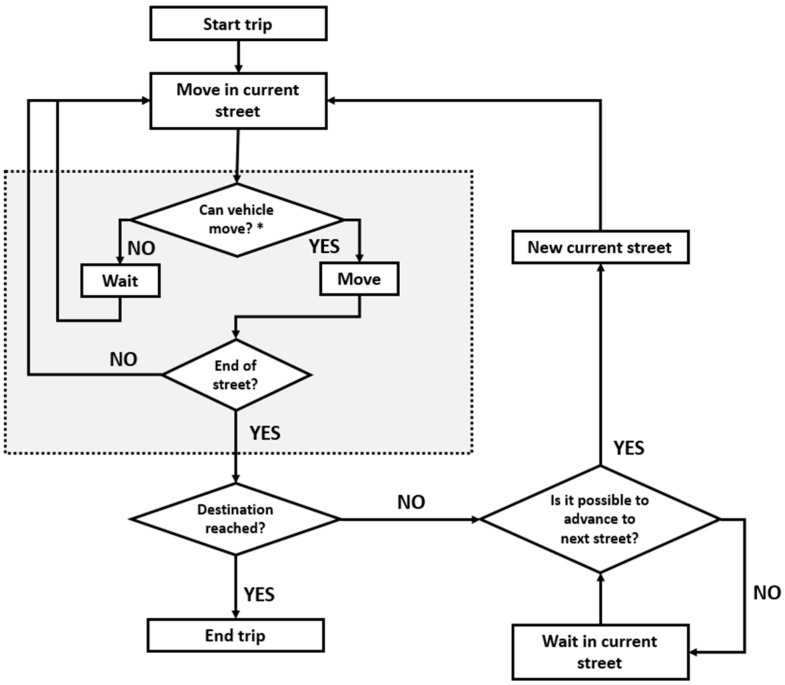
Vehicle movement simulation.

### 5.3. Comparison Metrics

#### 5.3.1. Route Efficiency

As the simulation developed acts in a discrete way, it was necessary to make some approximations in order to establish the route efficiency for a vehicle. We start from the fact that the number of times a vehicle must wait affects the quality of the route it is following. Efficiency in this case was defined in the following way:

(8) $$efficiency_{v}=\;\frac{number\;of\;moves\;}{number\;of\;moves+waits}$$

where the number of moves for a vehicle is determined by the total “slots” in the street or its capacity, and the waits are generated each time the vehicle cannot advance. This metric is proposed to directly be compared with the total distance traveled so that although the car takes a more costly route according to his distance, if there are not generated waits in the trip the efficiency is 1. 

#### 5.3.2. Traveled Distance

It is the total distance traveled by the vehicle from the origin node till the destination node:

(9) $$td=\;\underset{\left(i,j\right)\in R_{v}}{\sum }d_{\left(i,j\right)}$$

#### 5.3.3. Fairness Index

The fairness index is used as a compare metric to establish how the individual optimization for the vehicle’s route efficiency is related with the way the road infrastructure is being used. Following the Equation [2] used in Section 3.2, the fairness is calculated as follows:

(10) $$f_{\alpha }\left(k\right)=\frac{{\left[\sum^{}Use_{street_{i}}/Capacity_{street_{i}}\right]}^{2}}{S*\sum {\left(Use_{street_{i}}/Capacity_{street_{i}}\right)}^{2}}$$

where S is the total number of streets in the vehicular network and *k* the current state.

#### 5.3.4. Congestion

The average congestion is presented with the purpose of relate it with the efficiency and fairness obtained:

(11) $$\mu (k)=\frac{\sum^{}\left[\frac{Use_{\left(street_{i}\right)}}{Capacity_{\left(street_{i}\right)}}\right]}{S}$$

where S is the total number of streets in the vehicular network and *k* the current state.

## 6. Obtained Results

This section presents the results for each implementation in the set of evaluated scenarios. First, we present the control scenario results with the two different validation approaches described in Section 5.1.1: N vehicles and F vehicles. Later, we present the reference scenario results. The next section describes the result presentation.

### 6.1. Obtained Results Presentation

For each scenario we present two kinds of figures: those representing the general behavior of the corresponding metric, and those representing the result of comparing the metric value with the best value obtained. The description of each one is presented below.

#### 6.1.1. General Behavior 

**Figure 20:**
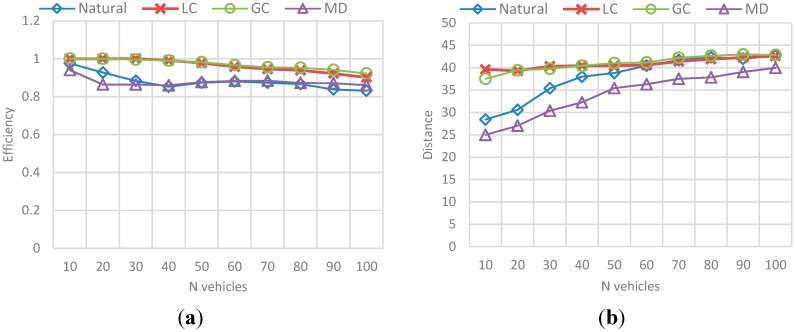
Behavior for route efficiency and trip distance in the N-vehicles scenario. (**a**) Mean efficiency value for each N-vehicles set; (**b**) Mean distance value for each N-vehicles set.

Figure 20, Figure 22, Figure 23, Figure 24, Figure 25, Figure 27, Figure 28, Figure 29 and Figure 31 present the values obtained for each implementation under the same scenario. The presented values correspond to (a) the mean value of each metric for a group of vehicles when the metric described is efficiency or distance; or (b) the specific value obtained for a state k of the simulation when the metric described is Fairness or average congestion. Specific details on the scenario are described in each one.

#### 6.1.2. Comparison against the Best Value

To get a better view of how good is an implementation with respect to another, a comparison percentage based on the best result for each metric is performed indicating how far from the best result is each implementation. When the best result should be the maximum value of the whole, this percentage is calculated as in Equation [12] and when the best value should be the minimum, the percentage is calculated as in Equation [13]:

(12) $$\%\;=\frac{\left(Best-Real\right)}{Best}$$

(13) $$\%\;=\frac{\left(Real-Best\right)}{Best}$$

In Equation [12], if the value obtained by the implementation is the maximum, the comparison percentage is in the line x = 0, and the following values are in the set of positive values above this line. This definition is used for *Fairness* and *Efficiency*. In the same way, in Equation [13], when the value obtained is the minimum, the comparison percentage is in line x = 0. This definition is used for *Distance* and *Congestion*. In both cases, as the percentage is bigger, the result is farther from the best value, and should be interpreted, for example, as *value X* is 30% worse than the best.

### 6.2. Control Scenario Results

#### 6.2.1. N Vehicles

In this scenario, 10, 20, 30, 40, 50, 60, 70, 80, 90 and 100 vehicles were moved along the vehicular network to examine each metric as the number of vehicles rises. Vehicles enter an empty network at the same time and simulation stops when all vehicles in the set arrive to the destination node, which means the network is empty after the final simulation step. 

##### (a) Efficiency and Distance 

**Figure 21:**
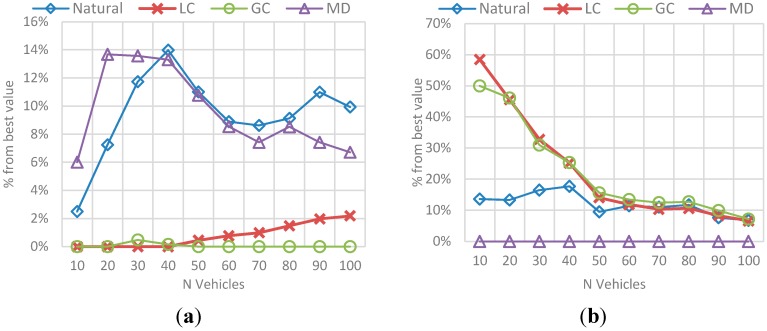
Best value comparison for route efficiency and trip distance in the N-vehicles scenario. (**a**) Percentage comparison against the best value obtained for efficiency; (**b**) Percentage comparison against the best value obtained for trip distance.

The results correspond to the average value for the total set of N vehicles passed during the simulation (*i.e.*, mean efficiency/total trip distance for 10 vehicles, 20 vehicles, and so on). The behavior in Figure 20 represents the obtained efficiency and trip distance, while the best-value comparison in Figure 21 represents the comparison percentage as described in Section 6.1.2. 

The main conclusions of this simulation with respect to the behavior of efficiency and distance are:

- The route efficiency in all implementations decreases as the set of vehicles in the network is bigger.
- The trip distance is affected by the amount of vehicles in the network and it increases as the number of vehicles grows, even for the MD solution.
- Even when the efficiency levels are always between 0.8 and 1 and the variability in the results is not very large, GC had a better result with respect to the other implementations, followed by LC.
- On the other hand, that implementation has one of the biggest average trip distance among the vehicles, which means it exists a trade-off between the travelled distance and the route efficiency.

The behavior of *Natural* and MD implementations is only differentiated by the trip distance. In the case of efficiency these two behave alike.

**Figure 22:**
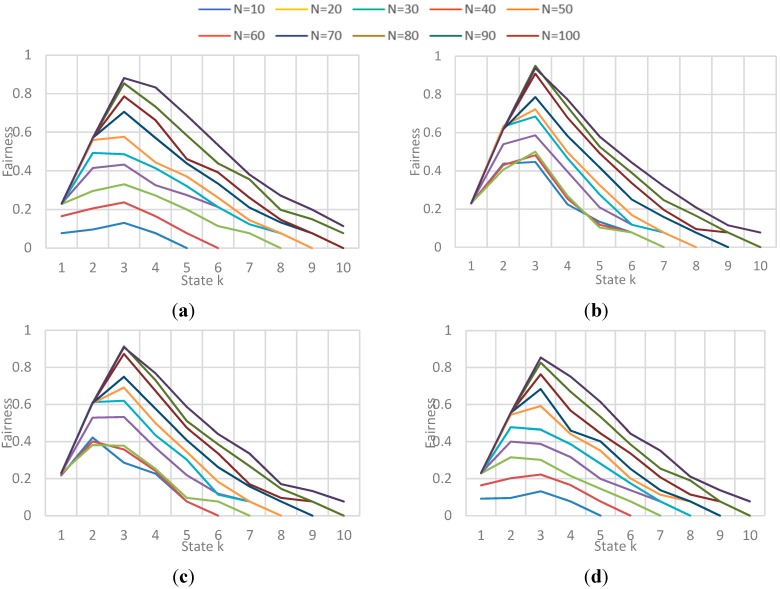
Obtained Fairness in a state k for each N-vehicle set with each implementation. (**a**) Fairness obtained for each N-vehicles with Natural behavior algorithm; (**b**) Fairness obtained for each N-vehicles with Local congestion algorithm; (**c**) Fairness obtained for each N-vehicles with Global Congestion algorithm; (**d**) Fairness obtained for each N-vehicles with Modified Dijkstra algorithm.

##### (b) Fairness and Congestion

**Figure 23:**
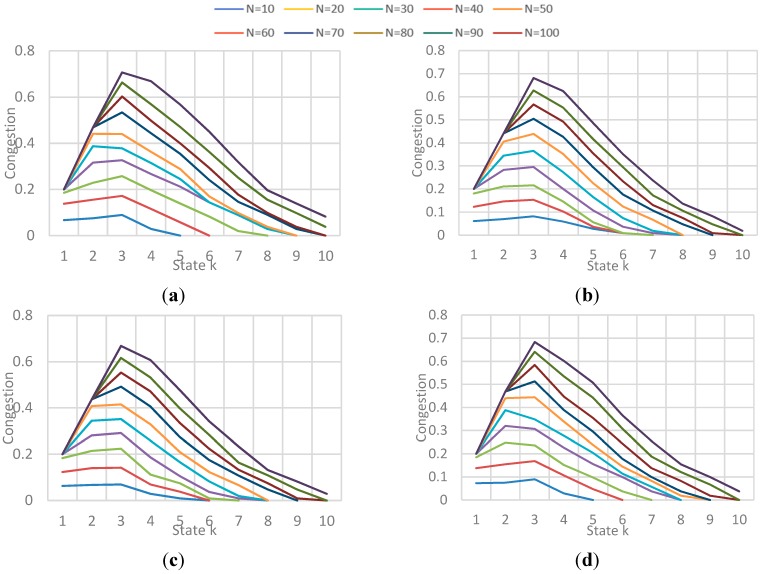
Obtained Average Congestion in a state k for each N-vehicle set with each implementation. (**a**) Avg. Congestion obtained for each N-vehicles with Natural behavior algorithm; (**b**) Avg. Congestion for each N-vehicles with Local congestion algorithm; (**c**) Avg. Congestion for each N-vehicles with Global Congestion algorithm; (**d**) Avg. Congestion for each N-vehicles with Modified Dijkstra algorithm.

The results correspond to the obtained value for fairness and congestion in a state k of the simulation. As the number N vehicles evaluated is not the same (10 to 100), the simulation could have more or less states, so it shows the results for all evaluated N vehicles presented in different graphs for each implementation. Figure 22 shows the behavior of Fairness for each implementation, and Figure 23 shows the behavior of average congestion for each implementation for each N-vehicles passed. 

The main conclusions of this simulation with respect to the behavior of fairness and congestion are:

- Fairness behavior and congestion behavior are very similar: as the maximum capacity of the streets is reached, the streets get saturated which result in a high congestion level implying that the use percentage of the streets is very similar.
- GC and LC implementations obtain the highest values in fairness when the congestion level is not high, that means the streets are being used better in those cases. Also, those implementations obtained the best values in efficiency, showing that exists a relation between the trip efficiency for each vehicle, and the use balance in the streets.

#### 6.2.2. F Vehicles

In this scenario, a constant number F of vehicles were incorporated to vehicular network in each simulation (k state). Vehicles enter an empty network and simulation stops when a number N of vehicles arrive at the destination node. This means that in contrast with the N-vehicle simulation scenario, there will be vehicles in the network after the final simulation step. Best value comparison for distance was not taken into account in the presented results.

##### (a) Efficiency and Distance

**Figure 24:**
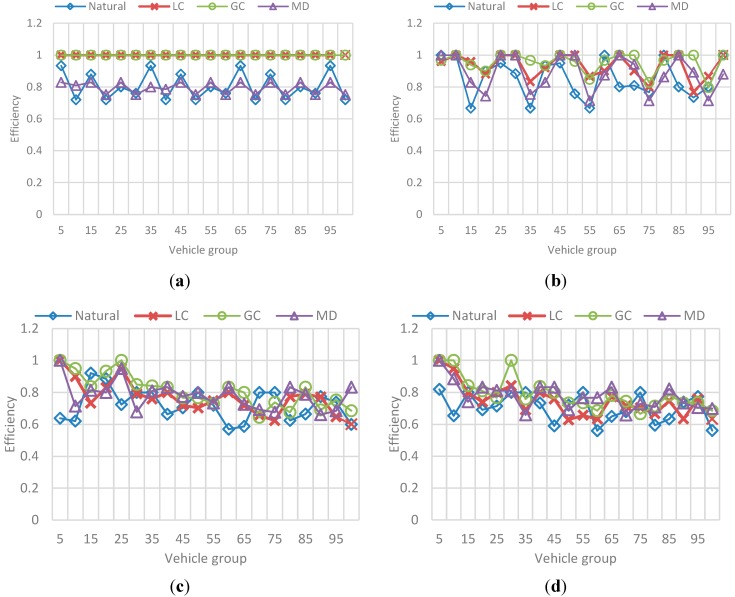
Behavior for route efficiency in the F-vehicles scenario. (**a**) Mean efficiency value for each 5-vehicles set when F is 10; (**b**) Mean efficiency value for each 5-vehicles set when F is 20; (**c**) Mean efficiency value for each 5-vehicles set when F is 30; (**d**) Mean efficiency value for each 5-vehicles set when F is 40.

**Figure 25:**
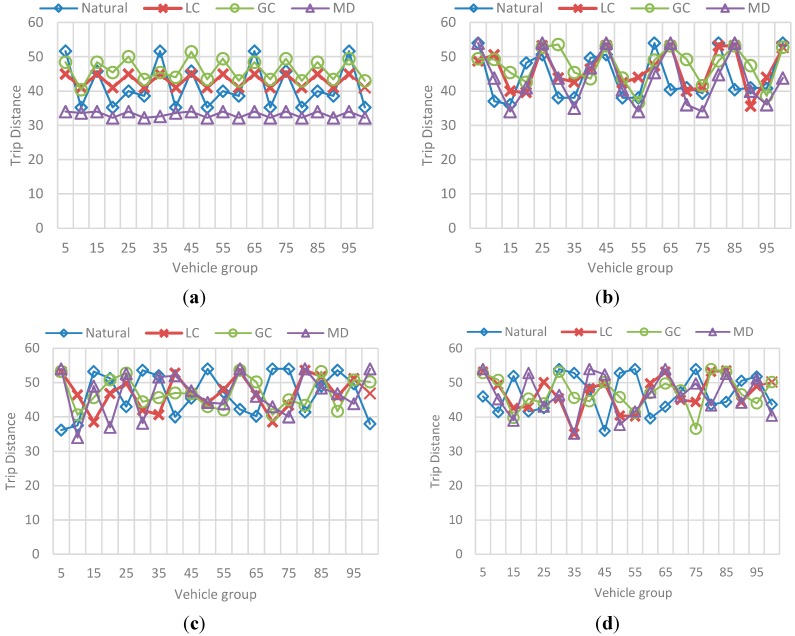
Behavior for trip distance in the F-vehicles scenario. (**a**) Mean trip distance value for each 5-vehicles set when F is 10; (**b**) Mean trip distance value for each 5-vehicles set when F is 20; (**c**) Mean trip distance value for each 5-vehicles set when F is 10; (**d**) Mean trip distance value for each 5-vehicles set when F is 20.

**Figure 26:**
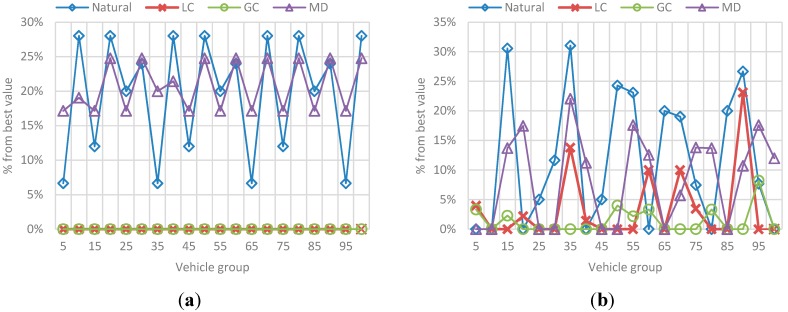

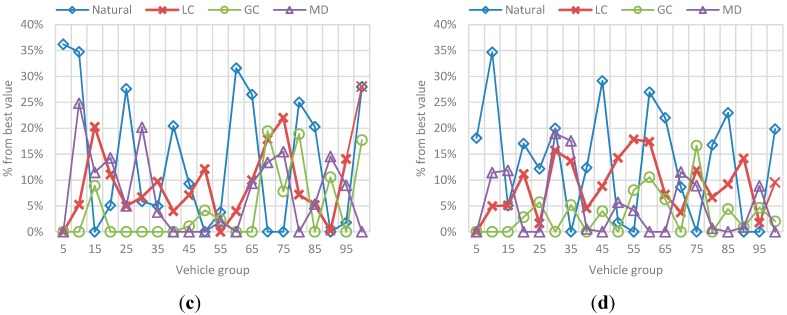
Best value comparison for efficiency in the F-vehicles scenario. (**a**) Percentage comparison against the best value obtained for efficiency for each 5-vehicles set when F is 10; (**b**) Percentage comparison against the best value obtained for efficiency for each 5-vehicles set when F is 20; (**c**) Percentage comparison against the best value obtained for efficiency for each 5-vehicles set when F is 30; (**d**) Percentage comparison against the best value obtained for efficiency for each 5-vehicles set when F is 40.

The results correspond to the average value for each group of 5 vehicles, in a sample of 100 vehicles of the total N vehicles passed during the simulation. Also, each metric is presented independently and contains the results for each selected *F*. Figure 24 represents the obtained efficiency and Figure 25 the obtained trip distance. Figure 26 represents the best-value comparison for efficiency. 

The main conclusions of this simulation with respect to the behavior of efficiency are:

- As the number *F* of vehicles increases, simulation results for efficiency in the previous simulation are replicated: the route efficiency in all implementations decreases as the set of vehicles in the network reaches the saturation value, in fact, for *F =* 30 and *F =* 40 there is not a great difference between efficiency values in the implementations.
- In addition, even when the efficiency values are similar GC have better results. In ontrast, this implementation have one of the worst trip distance value.
- Trip distance is again related with the efficiency showing a similar increasing/decreasing behavior compared with the efficiency behavior.

##### (b) Fairness and Congestion

**Figure 27:**
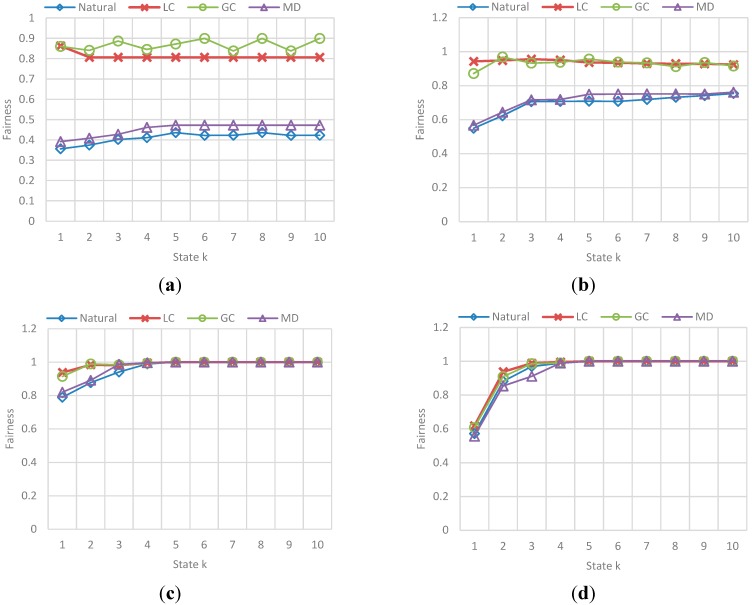
Obtained Fairness in a state k for each F-vehicle value. (**a**) Fairness obtained in a state k when F is 10; (**b**) Fairness obtained in a state k when F is 20; (**c**) Fairness obtained in a state k when F is 30; (**d**) Fairness obtained in a state k when F is 40.

**Figure 28:**
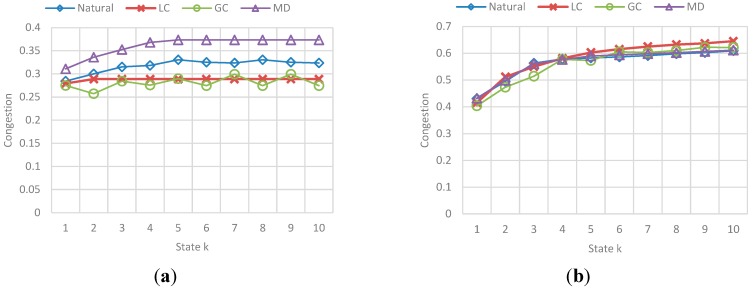

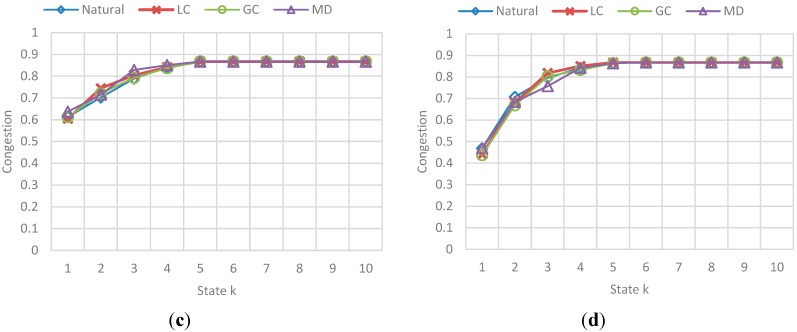
Obtained Average Congestion in a state k for each F-vehicle value. (**a**) Avg. congestion obtained in a state k when F is 10; (**b**) Avg. congestion obtained in a state k when F is 20; (**c**) Avg. congestion obtained in a state k when F is 30; (**d**) Avg. congestion obtained in a state k when F is 40.

The results for fairness and congestion correspond to a sample of the total states in the vehicular network and are presented in different graphics for each *F*. Obtained results are shown below:

The results correspond to the obtained value for fairness and congestion in a state k of a sample of states in the simulation. Figure 27 and Figure 28 show the behavior of Fairness and Congestion, that is, the value obtained for the state k, where k is one of 10 intermediate states of the simulation selected, for each *F*. Those 10 states where selected randomly with a uniform distribution to try to represent the initial, intermediate and final state of the vehicular network. 

The main conclusions of this simulation with respect to the behavior of fairness and congestion are:

- As the previous simulation, the GC and LC fairness is much better than *Normal* and SMD in low congestion levels of the network, while in high levels of congestion the fairness is pretty much the same. In addition, a relation between fairness and route efficiency exists.

### 6.3. Reference Scenario Results

**Figure 29:**
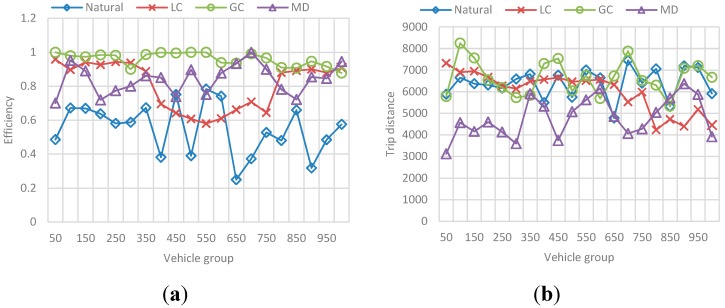
Behavior for route efficiency and trip distance. (**a**) Mean efficiency value for each 50-vehicles in a 100 vehicles sample; (**b**) Mean trip distance value for each 50-vehicles in a 100 vehicles sample.

**Figure 30:**
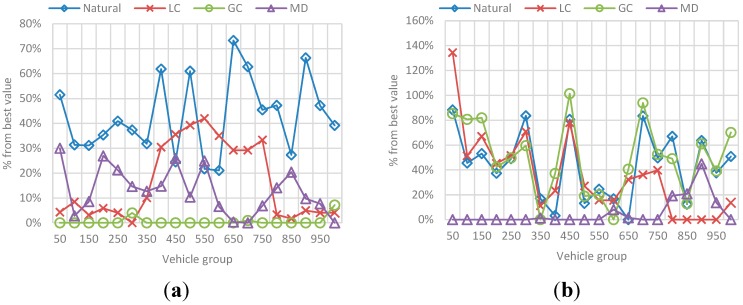
Best value comparison for efficiency and trip distance. (**a**) Percentage comparison against the best value obtained for efficiency; (**b**) Percentage comparison against the best value obtained for trip distance.

**Figure 31:**
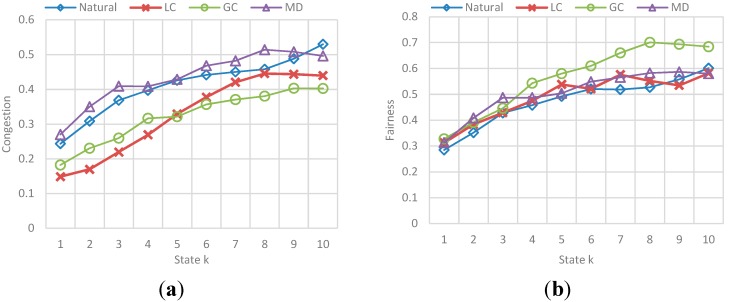
Behavior for Fairness and Congestion. (**a**) Fairness obtained in a state k; (**b**) Avg. congestion obtained in a state k.

In this simulation, a random number F of vehicles were incorporated to vehicular network in each simulation step. This number is in the interval [90, 600] where 90 is the average capacity of the set of ingress streets (the streets connected with the origin nodes). Vehicles enter an empty network and simulation stops when a number N of vehicles arrive at the destination node. The results in Figure 29 and Figure 30, correspond to the average value for each group of 50 vehicles, in a sample of 1000 vehicles of the total N vehicles that completed their trip. Figure 29 presents the obtained efficiency and Figure 30 presents the best-value comparison for efficiency and trip distance. In addition, the results in Figure 31 correspond to the behavior of Fairness and Congestion, that is, the value obtained for the state k, where k is one of 10 intermediate states of the simulation. Those 10 states where selected randomly with a uniform distribution to try to represent the initial, intermediate and final state of the vehicular network Obtained results are presented below:

As we can see, for a simulation in which both the source and destination of each vehicle, and the flow of vehicles entering the network in a step of the simulation is random, the results of control scenarios are replicated.

- It is appreciated that for GC, efficiency is more stable with the best results among the other implementations. Nevertheless, we can see again that the GC implementation that provides better efficiency is one of the implementation that has a greater distance, and the distance to DT usually better.
- Additionally, the average congestion in the simulation was not very big and the fairness values for implementations are very similar, however, GC has a better Fairness when the levels of congestion are no over the limit.
- For the Normal implementation, efficiency values are low (up to 60%–70% worse compared with the best value) and distance is not the best, so this implementation really does not offer any advantage over the others.

## 7. Conclusions

The Internet of Things allows the integration of elements in such a way that old problems can be addressed in new ways, especially for cases where the need of dealing with a constant changing environment becomes a priority. It was proved that the vehicular congestion problem, where it becomes a challenge provide a mechanism capable of dealing with the high dynamism of this parameter is constantly altering the global state of a vehicular network can be supported by this new paradigm.

A routing mechanism based on communication V2I in which nodes able to interact with vehicles was developed and compared against other approaches, namely, a heuristic approach based on shortest path distance, an approach aware of local parameters only, and an approach in which the decision regarding the route did not rely in any supporting communication. The results shown prove that: (1) the decision should not only be aware of the congestion state and act in a reactive manner to it, but should be oriented to achieving a lower congestion in every moment; (2) the information about local state of congestion is not enough for achieving an optimal efficiency and better results in a vehicle’s trip, so it is necessary to communicate the global information to every element in such a way a proper decision is made; and (3) the lack of any supporting mechanism in search of the best route always leads to worse results.

The proposed solution had the best performance for both efficiency and fairness, in scenarios with different characteristics. Therefore, it is a more robust solution and applied in a real context it is likely to generate a benefit in the route taken by a vehicle. In addition, the relation shown between fairness and efficiency and the higher results of the former obtained by the proposed solution imply that vehicles are using the vehicular infrastructure in a more efficiently way. That’s why in the future work is the development a V2I and V2V communication mechanism that allows a global optimization of the congestion not for individual trips of the vehicles, but for the whole vehicular network.
